# Tracking the Trajectory of Functional Humoral Immune Responses Following Acute HIV Infection

**DOI:** 10.3389/fimmu.2020.01744

**Published:** 2020-08-07

**Authors:** Madeleine F. Jennewein, Jennifer Mabuka, Cassidy L. Papia, Carolyn M. Boudreau, Krista L. Dong, Margaret E. Ackerman, Thumbi Ndung'u, Galit Alter

**Affiliations:** ^1^Ragon Institute of Massachusetts General Hospital, Massachusetts Institute of Technology and Harvard University, Cambridge, MA, United States; ^2^Africa Health Research Institute, Durban, South Africa; ^3^HIV Pathogenesis Programme, Doris Duke Medical Research Institute, Nelson R. Mandela School of Medicine, University of KwaZulu-Natal, Durban, South Africa; ^4^Thayer School of Engineering, Dartmouth College, Hanover, NH, United States; ^5^Max Planck Institute for Infection Biology, Berlin, Germany; ^6^Division of Infection and Immunity, University College London, London, United Kingdom

**Keywords:** HIV, antibodies, non-neutralizing, polyfunctionality, acute infection

## Abstract

Increasing evidence points to a role for antibody-mediated effector functions in preventing and controlling HIV infection. However, less is known about how these antibody effector functions evolve following infection. Moreover, how the humoral immune response is naturally tuned to recruit the antiviral activity of the innate immune system, and the extent to which these functions aid in the control of infection, are poorly understood. Using plasma samples from 10 hyper-acute HIV-infected South African women, identified in Fiebig stage I (the FRESH cohort), systems serology was performed to evaluate the functional and biophysical properties of gp120-, gp41-, and p24- specific antibody responses during the first year of infection. Significant changes were observed in both the functional and biophysical characteristics of the humoral immune response following acute HIV infection. Antibody Fc-functionality increased over the course of infection, with increases in antibody-mediated phagocytosis, NK activation, and complement deposition occurring in an antigen-specific manner. Changes in both antibody subclass and antibody Fc-glycosylation drove the evolution of antibody effector activity, highlighting natural modifications in the humoral immune response that may enable the directed recruitment of the innate immune system to target and control HIV. Moreover, enhanced antibody functionality, particularly gp120-specific polyfunctionality, was tied to improvements in clinical course of infection, supporting a role for functional antibodies in viral control.

## Introduction

Several lines of evidence suggest that the initial serum cellular and antibody responses to HIV infection shapes the course of disease (1). Early cellular responses, primarily NK and T cells (2–6), have been linked to enhanced viral control, the establishment of a lower viral set-point, and slower progression to AIDS (1). While strain-specific neutralizing antibodies and binding antibodies emerge within months of infection, the virus is quickly able to evade and escape from these responses (1, 7–9). It takes several years of infection for broadly neutralizing antibodies to evolve (10). However, while antibody levels seem to increase with progression, rather than control (11), emerging data suggest that qualitative differences (subclass, function, etc.)—rather than the overall levels of antibodies—are uniquely functionally optimized in non-progressors (12). Specifically, individuals who spontaneously control HIV harbor elevated levels of functional antibodies, able to leverage NK cell cytotoxicity, monocyte phagocytosis (13, 14), neutrophil function, and complement activation (2, 3, 15–17). Interestingly, while specific HLA class I alleles, essential for T cell mediated immunity, account for viral control in ~15% of spontaneous controllers of HIV (18); functional antibodies evolve in a larger fraction of “controllers” in an HLA-independent manner, potentially pointing to a globally relevant harness-able immune mechanism of viral control (19).

While gp120-specific functional antibody responses have been deeply investigated due to their proposed role in both neutralization and antibody dependent cellular cytotoxicity (ADCC), gp41-specific responses appear earliest following infection (1) and mounting evidence points to a potentially critical biomarker role for p24-specific functional antibodies in spontaneous control of HIV (20–24). Beyond ADCC, antibodies are able to drive antibody dependent phagocytosis (ADCP), antibody dependent complement deposition (ADCD), and ADCC, key factors in the control and prevention of HIV (25–27). Specifically, spontaneous controllers of HIV develop polyclonal, gp120-specific, pools of antibodies that are able to drive polyfunctional antibody profiles (16, 28). Furthermore, several of these antibody functions have been associated with viral control in non-human primates (29–31) and have been linked to preventing infection in mice (32). In humans, these functions have been associated with reduced mother-to-child transmission (11, 33), and are enriched among individuals able to spontaneously control infection (2, 3, 16, 28). Moreover, specific antibody effector functions have been linked to reduced risk of infection in both humans (34, 35) and non-human primate vaccine studies (36–38), collectively pointing to the critical nature of antibody effector functions in both the control and prevention of infection. While many studies have investigated the functional profile of specific monoclonals (39–42), little is known about the overall evolution of antibody effector functions within the polyclonal immune response across different antigen specificities in early infection.

To further to define the profile of the early functional antibody response to HIV in the serum, the biophysical and functional profile of gp120-, gp41-, and p24-specific antibodies were assessed in ten hyper-acutely infected women. These women were recruited before early treatment became the standard of care in South Africa, when treatment was based on CD4 cell number, and thus these women were untreated over the first year of infection. Rapid antibody subclass, glycosylation, and effector functional evolution was observed in the first few months of infection. However, these changes occurred in an antigen-specific manner. Interestingly, we show that enhanced antibody polyfunctionality, rather than any single function or antibody Fc-profile change, and particularly polyfunctionality of the gp120-specific response, is correlated with improved viral control and thus improved clinical course.

## Methods

### FRESH Cohort

Peripheral blood samples were collected from 10 women enrolled in the FRESH cohort in Kwa-Zulu-Natal, South Africa who were acutely infected with HIV [(43); Supplementary Table 1]. The ten participants were identified in Fiebig stage I a median of 4 days after a negative plasma RNA (range 3–21) and remained off anti-retroviral therapy during the duration of the study, as the participants' clinical course had yet to reach the national eligibility criteria for treatment at the time (43). Viral load and percentage of CD4+ T cells were measured at each of visit. The study was reviewed by the Partners Institutional Review Board and approved by the Partners Human Research Committee and the Biomedical Research Ethics Committee of the University of KwaZulu-Natal. All subjects provided written informed consent.

### Sample Preparation

Peripheral blood plasma samples were collected at 1, 3, 9, and 12 months of infection. Plasma was separated from whole blood and cryopreserved. Prior to analysis, all plasma was then heat inactivated at 56°C for 1 h to eliminate complement activated immune complex activity that may confound analysis. Plasma samples were then spun at 16,000 × g for 10 min, and supernatants were isolated and stored at −80°C until use.

### Proteins

Antibody responses to three clade C HIV antigens were measured: monomeric HIV gp120 Clade C Du151, monomeric HIV gp41 ectodomain, and HIV p24 Clade B/C CN54, for which early antibody responses arise, and are often diagnostic, to track overall antibody responses over time. All proteins were obtained from Immune Technology Corp (NY, USA).

### Phagocytosis Assays

#### Antigen Coupling to Beads

Antigens were biotinylated with EZ-Link NHS-LC-biotin (ThermoFisher, MA, USA) according to manufacturer's instructions. Excess unbound biotin was removed using a Zeba Spin desalting column (ThermoFisher), and proteins were re-suspended in PBS at 1 mg/mL. Ten micrograms of biotinylated proteins were coupled individually to 10 μl of 1 μm yellow-green fluorescent, neutravidin-coated microspheres (FluoSpheres, Life Technologies, CA, USA) by incubating at 37°C for 2 h. Beads were washed twice with PBS-5% BSA to block. Beads were resuspended in a final volume of 1 mL PBS-0.1% BSA and stored at 4°C in the dark for up to 1 week.

#### Formation of Immune Complexes

Ten microliters of protein-coated beads were incubated with 10 μl of heat-inactivated plasma diluted at 1:100 in PBS in a 96-well U-bottom culture plate. HIV positive plasma was used as a positive control, and PBS was used as a negative control. Following a 2-h incubation at 37°C, 5% CO_2_, the immune complexes were washed in PBS and incubated with either THP-1 cells or neutrophils as described below.

#### Monocyte Antibody-Dependent Cellular Phagocytosis (ADCP) Assay

The monocyte ADCP assay was adapted from a Good Clinical Laboratory Practice (GCLP) qualified assay that measures immune complex uptake into monocytes (44) that has been previously associated with protection against SIV/SHIV in non-human primates (38, 45). Briefly, immune complexes were incubated with 25,000 THP-1 cells (ATCC, VA, USA) per well at a concentration of 1.25 × 10^5^ cells/ml in R10 (RPMI with 1% Pen/Strep, 1% HEPES, 1% L-glutamine and 10% FBS) for 16 h at 37°C, 5% CO_2_. Following the incubation, cells were fixed in 4% paraformaldehyde. Data were collected on a BD LSR II flow cytometer (BD Biosciences, CA, USA) equipped with FACS Diva software. Flow cytometry data were analyzed using Flowjo (TreeStar, OR, USA). Negative or unstimulated controls were used to set gates. A phagocytosis score was calculated as the percentage of bead positive cells, multiplied by geometric mean fluorescence intensity of bead positive cells, divided by 10,000. Value for HIV- plasma was subtracted from all samples to normalize for background. Data are reported as the mean of two replicates.

#### Antibody-Dependent Neutrophil Phagocytosis (ADNP) Assay

The ADNP assay was performed as previously described GCLP qualified assay that quantifies immune-complex uptake into cells (46) that was previously associated with protection from SIV infection in non-human primates (38). Briefly, whole blood was collected in ACD tubes. Granulocytes were isolated by lysing erythrocytes with ACK lysis buffer (Thermo Fisher Scientific, MA, USA) for 5 min before precipitation by centrifugation. Granulocytes were washed twice with PBS and were resuspended at 2.5 × 10^5^ cells/ml in R10 and 50,000 cells per well were incubated with immune complexes for 1 h at 37°C, 5% CO_2._ Neutrophils were stained with anti-CD66b (Biolegend, San Diego, CA), and cells were fixed with 4% paraformaldehyde prior to flow cytometry. Phagocytosis scores were calculated as above in the ADCP assay.

### Antibody-Dependent Complement Deposition

The ADCD assay was adapted from a GCLP qualified assay (47). Specifically, HIV antigens were biotinylated and coupled to 1 μm red fluorescent neutravidin microspheres (Fluospheres, ThermoFisher) as described above. Immune complexes between plasma sample and beads were formed as described above and incubated for 2 h. Lyophilized guinea pig complement (Cedarlane, Canada) was rehydrated in ice cold H_2_O and diluted to 2% complement in gelatin veronal buffer with Magnesium and calcium (Boston Bioproducts, MA, USA). Following the 2-h incubation, plate was washed and 200 μl per well of complement was added and incubated at 37°C, 5% CO_2_ for 30 min. After incubation beads were washed twice in 15 mM EDTA (Fisher Scientific). Complement deposition was detected with FITC-conjugated, goat anti-guinea pig complement C3 (MP Biomedicals, CA, USA) for 30 min at RT. Beads were resuspended in PBS. Samples were analyzed on the iQue Screener PLUS platform (Intellicyt, NM, USA). Data was analyzed using ForeCyt software (Intellicyt) and recorded as median fluorescent intensity of FITC. Value for HIV- plasma was subtracted from all samples to normalize for background.

### NK Cell Activation Assay

The NK cell activation assay measures CD107a, IFN-γ, and MIP-1β levels as a robust surrogate for direct measurement of antibody-dependent cellular cytotoxicity, as previously described (48, 49). ELISA plates were prepared by coating plates with 50 μL of protein at 1 μg/mL in PBS followed by a 2 h incubation 37°C 5% CO_2_. Plates were then washed and blocked with PBS-5% BSA overnight at 4°C. Plates were washed with PBS, and 50 μL plasma was plated at a 1:10 dilution in PBS. HIV positive plasma was used as a positive control, and PBS was used as a negative control. Plates were incubated at 37°C 5% CO_2_ for 2 h for immune complex formation. During this time, NK cells were isolated from healthy adult buffy coats drawn the previous day. NK cell isolation was performed with RosetteSep NK Cell Enrichment Kit (Stem Cell Technologies, Canada) per the manufacturer's instructions. Purified NK cells were separated by density centrifugation, washed twice with PBS and used immediately. The isolated NK cells were then added to each well at a concentration of 2.5 × 10^5^ cells/mL, 50,000 cells per well, in R10 in the presence of anti-CD107a (BD Biosciences, CA, USA), Brefeldin A (2.5 μg/mL; Biolegend, CA, USA), and GolgiStop (BD Biosciences, CA, USA) and incubated at 37°C, 5% CO_2_ for 5 h. After the incubation, cells were stained with anti-CD3, anti-CD56, and anti-CD16 antibodies (all BD Biosciences, CA, USA). Cells were fixed and permeabilized using the BD Fix/Perm kit (BD Biosciences, CA, USA), and intracellular staining was performed with anti-IFNγ and anti-MIP-1β (both BD Biosciences, CA, USA). Data were collected on a BD LSR II flow cytometer (BD Biosciences, CA, USA) equipped with FACS Diva software. Flow cytometry data were analyzed using Flowjo (TreeStar, OR, USA). Negative or unstimulated controls were used to set gates. Data were reported as the percentage of NK cells positive for a given marker minus the mean of the protein-matched PBS-only control.

### Antigen-Specific Antibody Isotype and Subclass Analysis

Antigen-specific IgG isotype and subclass levels were measured by multiplexed Luminex assay as described in (50). HIV antigens were coupled to Magplex microspheres (Luminex corporation, TX, USA) using carboxyl chemistry. Microspheres were first activated with 100 mM monobasic sodium phosphate, pH 6.2 (Sigma-Aldrich, CA, USA) in the presence of 50 mg/mL EDC and 50 mg/mL sulfo-NHS (both Thermo Fisher Scientific, MA, USA). Beads were then washed in 0.05 M 2[N-Morpholino] ethanesulfonic acid (MES) pH 5.0 (Boston BioProducts, MA, USA) and incubated with antigen for 2 h at a ratio of 25 μg of antigen to 5 × 10^6^ microspheres. The beads were blocked with PBS-TBN (PBS-0.1%BSA, 0.02% Tween 20, 0.05% Azide, pH 7.4) for 30 min. Then, beads were washed with 0.05% PBS-Tween 20 and blocked in PBS-2% BSA for 2 h. After washing with 0.05% PBS-Tween 20, the beads were resuspended in PBS and stored at 4°C in the dark. Plasma samples were then diluted in luminex wash buffer at 1:100 (IgG1-4, IgA1, IgA2, and IgM) or 1:500 (Total IgG and IgG1) (PBS-0.05% BSA-0.001% Tween-20) in a 384-well plate with duplicates for each isotype or subclass in (PBS-0.05% BSA-0.001% Tween-20) and incubated with coupled microspheres for 2 h. Beads were then washed in Luminex wash buffer and then incubated with PE-labeled secondaries to Total IgG, IgG1, IgG2, IgG3, IgG4, IgA1, IgA2, or IgM (Southern Biotech, AL, USA). Samples were washed again and resuspended in xMAP sheath fluid (Luminex corporation, TX, USA). Samples were analyzed on a Bioplex 3D system. Data were calculated as the median fluorescent intensity of PE.

### Glycosylation Analysis

Antigens were biotinylated and coupled to streptavidin magnetic beads (New England Biolabs) at a ratio of 2.5 μg of protein to 25 μl of beads per each sample for 30 min and then washed. Two hundred microliter of plasma samples were incubated with non-antigen-coated neutravidin beads to remove non-specific binding for 30 min. Plasma was then removed and added to antigen-coated beads and incubated for 1 h at 37°C. To isolate the Fc N-glycan, IDEZ (New England Biolabs) was used to cleave off the Fc. One microliter of IDEZ was added to the antibody-bound beads in a total volume of 20 μl of PBS and incubated at 37°C for 1 h. The cleaved Fc fragments were deglycosylated, and fluorescently labeled using a GlycanAssure APTS kit (ThermoFisher Scientific) according to manufacturer's instructions. Briefly, the Fc fragment was deglycosylated with PNGase, purified on glycan-binding beads, then fluorescently labeled with 8-Aminopyrene- 1,3,6-Trisulfonic Acid (APTS) via reductive amination, and then washed again using glycan-binding beads. Glycans were analyzed on a 3500xL genetic analyzer. Glycan fucosyl and afucosyl libraries (Prozyme) were used to assign 24 discrete glycan peaks using GlycanAssure software (ThermoFisher). Data were reported as percentages of total glycans for each of the glycan peaks.

### Antigen-Specific Antibody Fc Receptor Binding

Antigen-specific binding to Fc receptors was assessed similarly to antibody isotype and subclass analysis, as previously described (50). HIV antigen-coupled microspheres as described above were used. Plasma samples were diluted in Luminex wash buffer at either 1:250 of 1:1,000 and were detected with recombinant Fc-receptor detector tetramers; FCGR2A-H (high affinity variant), FCGR2A-R (low affinity variant), FCGR2B, FCGR3A-V (high affinity variant), FCGR3A-F (low affinity variant), and FCGR3B (51) or C1q (Sigma-Aldrich) in duplicate. Samples were washed again and resuspended in xMAP sheath fluid (Luminex corporation). Samples were analyzed on a FlexMap3D instrument (Luminex) and raw data were reported as median fluorescent intensity values.

### Antibody Avidity

Avidity was assessed using an ELISA assay including a urea dilution (52). 96-well Immulon plates (ThermoFisher) were coated with 100 μL of 250 ng/mL of each HIV antigen in PBS overnight at 4°C. The next day plates were washed in PBS-0.05% Tween-20 and blocked with PBS-2% BSA for 2 h at room temperature. Samples were diluted in PBS, plated in duplicate on ELISA plates, and incubated for 2 h at room temperature. Plates were washed again, and PBS-0.05 Tween-20 was added to half the plates and 8 M Urea (Sigma Aldrich) was added to the treated replicates. After 5 min of incubation on a shaker, plates were flicked. This was repeated for a total of three washes with 8 M urea. Plates were washed with PBS, and antibody binding was detected with goat anti-human IgG-HRP (Bio-Rad, CA, USA). Developing solution was prepared by dissolving an OPD tablet (ThermoFisher) in 11 mL of PBS with 4.4 μL of hydrogen peroxide. Samples were developed, and the reaction was quenched with 2 N sulfuric acid. ELISA plates were read on a Tecan Infinite M1000 Pro at 490 nm with reference at 570 nm.

### Univariate Statistical Analysis

Data on radar plots was z-score normalized individually along each radar to an average of 0 with a standard deviation of 1. A one-way ANOVA was used to examine differences for across the four timepoints for antigen-specific functions, glycosylation, and subclass. Heatmaps correlating functions and subclass or functions and glycosylation were evaluated with spearmen correlations, showing the Spearman r value and stars indicate significance. Polyfunctionality was evaluated by performing a median split, with those values that fall above the median assigned as having the function, and those below the median as not having the function. Correlations between clinical characteristics and functionality or change in polyfunctionality were evaluated using Spearman correlations. A Bonferroni correction was performed to correct for multiple comparisons. *P*-values are all two-sided. Statistical analyses were conducted using GraphPad Prism.

### Principal Component Analysis (PCA)

PCA models were constructed to compare antigen-specific glycosylation and to compare the entire antigen-specific response between antigen-specificity and timepoint. For the glycan PCA the input variables were the 24 glycan peaks. For the model analyzing antigen specificity all antigen-specific data—avidity, subclass/isotype, Fc receptor binding, non-neutralizing functions, and glycans were included in the PCA analysis. Each antigen-specific response was treated as an individual data point and all values were calculated as deltas (3M-1M, 9M-3M, and 12M-9M). For both models, all variables were centered and scaled to a standard deviation of 1 and PCAs were constructed in Matlab (Mathworks, Natick, MA, USA).

### LASSO-Partial Least Squares Regression (PLSR)

Least absolute shrinkage and selection operator (LASSO) regression analysis was used to define the minimal set of features that separated the samples by timepoints, as previously described (53). Briefly, PLSR was used to model the separation along time course achieved by the LASSO-selected features when combined into latent variables, which linearly combine the features to describe the most variance in outcome along a two-dimensional plot. The model was orthogonalized so that Latent Variable 1 (LV1) captures the variance in timepoint while other variables are orthogonal to LV1. To test the robustness of the model, the PLSR analysis included 1,000 repetitions of 10-fold cross validation to obtain a CV *r*-value of 0.96. Variable importance in projection (VIP) scores were calculated, as a weighted sum of squares of the PLS weights, which summarize the importance of the various features to the PLSR model.

## Results

### Functional Antibodies Develop Over the First Year of Infection

To begin to understand the evolutionary kinetics of the functional serum antibody response to HIV infection, and to move beyond the assessment of functionality in HIV-monoclonals, ten acutely infected women with HIV (identified in Fiebig stage I), who were participants in the FRESH cohort, were followed over the first year of infection [(43); Table 1 and Supplementary Figure 1]. Plasma samples from 1 month (at a time when binding antibodies become detectable), 3, 9, and 12 months of infection were comprehensively profiled for their antibody response to infection, creating a detailed picture of the longitudinal development of non-neutralizing antibody responses including ADCP, ADNP, ADCD, and NK activation (as a surrogate for ADCC). These assays are designed to assess the contribution of the polyclonal immune response to each function, primarily focused on the evolution of the IgG response after infection (41).

**Table 1:** Clinical characteristics of study participants.

**Participant**	**Days since** **negative viral load**	**Log_**10**_ copies** **per mL** **at detection**	**Fiebig stage** **at detection**	**Peak viral** **load, Log**_**10**_ **copies per mL**	**Days to** **peak viral load**	**Started** **cART**	**Days to** **treatment initiation**
1	4	5.03	I	7.11	7	No	
2	4	2.67	I	7.04	7	Yes	1,589
3	3	4.56	I	7.48	11	Yes	347
4	4	3.29	I	6.91	10	Yes	310
5	4	2.2	I	7.72	10	Yes	809
6	21	5.94	III	8.29	4	Yes	700^*^
7	3	2.97	I	7.15	7	Yes	427
8	4	1.99	I	6.6	17	Yes	456
9	3	3.23	I	6.99	8	Yes	330^*^
10	4	4.84	I	7.76	7	Yes	724
Median	4	3.26		7.13	7.5	Yes	456

* *Approximate*.

**Figure 1:**
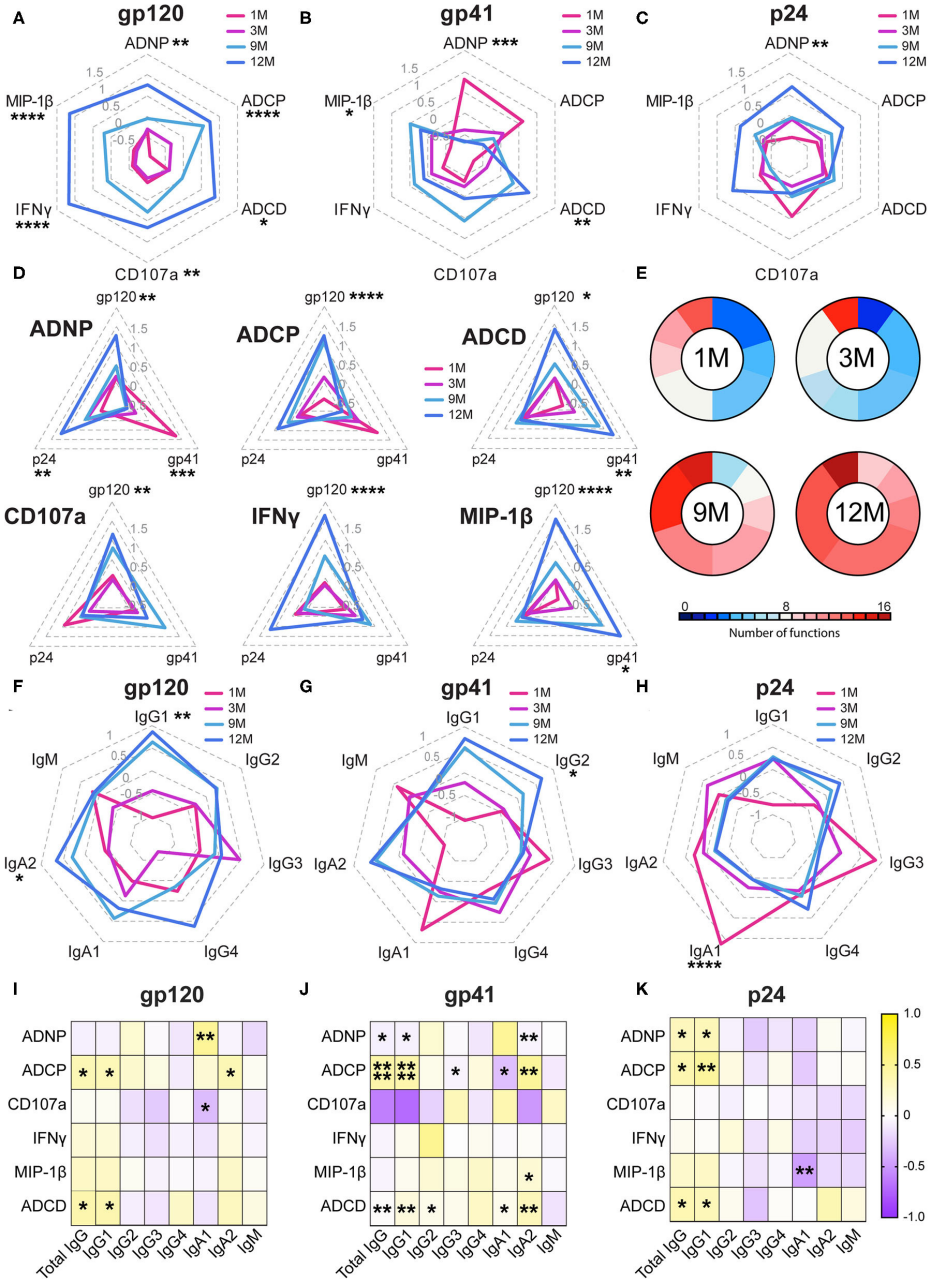
Non-neutralizing functions develop during acute infection. **(A–C)** Radar plots show the relative antigen-specific non-neutralizing functions elicited at 1-month (pink), 3 months (purple), 9 months (light blue), and 12 months (dark blue) of infection for gp120- **(A)**, gp41- **(B)**, and p24- **(C)** specific responses. Each function displayed is normalized to an average of 0 and a standard deviation of 1, and the average of the 10 subjects is graphed along the radars. Radars are labeled from 1.5 to−0.5 in light gray. **(D)** Radar plots show the gp120-, gp41-, and p24- specific response for each of the six functions measured: ADNP, ADCP, ADCD, CD107a expression, IFNγ expression, and MIP-1β expression. Titer by Luminex displayed are normalized to an average of 0 and a standard deviation of 1 and the average of the 10 subjects is graphed along the radars. Radars are labeled from 1.5 to −0.5 in gray **(E)**. The pie charts show polyfunctionality scores at each timepoint. The presence of each function was determined by a median split. The size of each slice represents the number of subjects who have a given number of functions at each timepoint and colored according to the number of functions. **(F–H)** Radar plots show the relative titers of each subclass/isotype for antigen-specific antibodies. Radars are labeled from 1 to −1 in gray. **(I–K)** Heatmaps show the r value of Spearmen correlation between function and subclass/isotype across all four timepoints. Statistics for radar plots were evaluated using one-way ANOVA. Heatmap was generated with spearman correlation. *p < 0.05, **p < 0.01, ***p < 0.01, and ****p < 0.001.

While neutralizing antibodies were not detected in any of these women (54), functional responses and antibody biophysical profiles (subclass/isotype, avidity, Fc-glycosylation, and Fc receptor binding) were all captured against gp120, gp41—for which responses emerge earliest (1)—and the internal gag antigen (p24), that are used diagnostically due to their early evolution and are associated with enhanced viral control (20–23). Functional responses to all three antigens showed a marked increase during infection, however this evolution varied in an antigen-specific manner (Figures 1A–C and Supplementary Figure 2). Specifically, while gp120-specific antibodies showed the greatest magnitude of change in their functions over the year, with all functions increasing, gp41-specific responses exhibited a distinct evolutionary profile. Early in infection, gp41-specific antibodies, thought to initially be cross reactive with the gut microbiome (55, 56), induced robust neutrophil and monocyte phagocytosis, which declined over the first year, while the NK cell and complement-activating response evolved oppositely, increasing over the year (Figure 1B). Interestingly, p24-specific responses showed a similar pattern to gp120; very early antibody-mediated activation decreased, with a concomitant evolution of all functions (Figure 1C). However, individually, the functions exhibited distinct profiles across the three antigens (Figure 1D), with gp120 and p24-specific antibodies showing more similar evolutionary profiles compared to gp41. These data point to distinct functional evolutionary profiles linked to antigen specificities, indicating discrete and independent regulation of the antigen-specific responses during early infection. Despite these antigen-specific differences across antigens, collectively, polyfunctionality increased over the first year of infection, tracking with the maturation of the humoral immune response (Figure 1E).

### Coordination Between Functional Evolution and Antibody Levels

To begin to define the biophysical changes in the humoral immune response that may underlie functional evolution, we next profiled the overall changes in antibody isotype and subclass selection over the course of the first year of infection (Figures 1F–H and Supplementary Figure 3). As expected, IgM and IgG3 responses to all three antigens dominated early in infection across all antigens, but regressed with the progressive maturation of the humoral immune response, in line with their order in the antibody locus (57). While the gp120-specific response at 1 month was dominated by IgM antibodies, the gp41 and p24 specific humoral immune responses accessed IgA, IgM as well as early IgG3 responses as early as 1 month following infection (Figures 1F–H). Moreover, gp120 and gp41-specific IgG1 antibody levels consistently increased in magnitude over the course of the first year of infection (Figures 1F,G). p24-specific antibody responses hit maximum levels by 3 months post-infection (Figure 1H) and gradually switched to a profile dominated by the less functional IgG2 and IgG4 subclasses (Figure 1H). Collectively, these data highlight different evolution patterns among antigen-specificities over time.

To understand the relationship of these antibody subclass changes to functionality, correlations between functions and subclasses across all timepoints were calculated (Figures 1I,J). Here, the correlations between function and subclass were largely consistent across all three antigens, highlighting conserved biophysical:function relationships irrespective of the antigen-specificity and functional evolutionary profile. ADCP, ADNP, and ADCD seemed to be most closely tied to subclass IgG, IgG1, IgA1, and IgA2 titers (Figures 1I–K). This is consistent with the predominance of these antibody classes/subclasses within the blood (57). However, relationships with NK cell mediated CD107a, IFNγ, and MIP-1β were less clear. Thus, these data suggest that similar biophysical changes drove antibody effector functions irrespective of antigen specificity or evolutionary pattern.

### Antibody Glycosylation Changes During Infection

**Figure 2:**
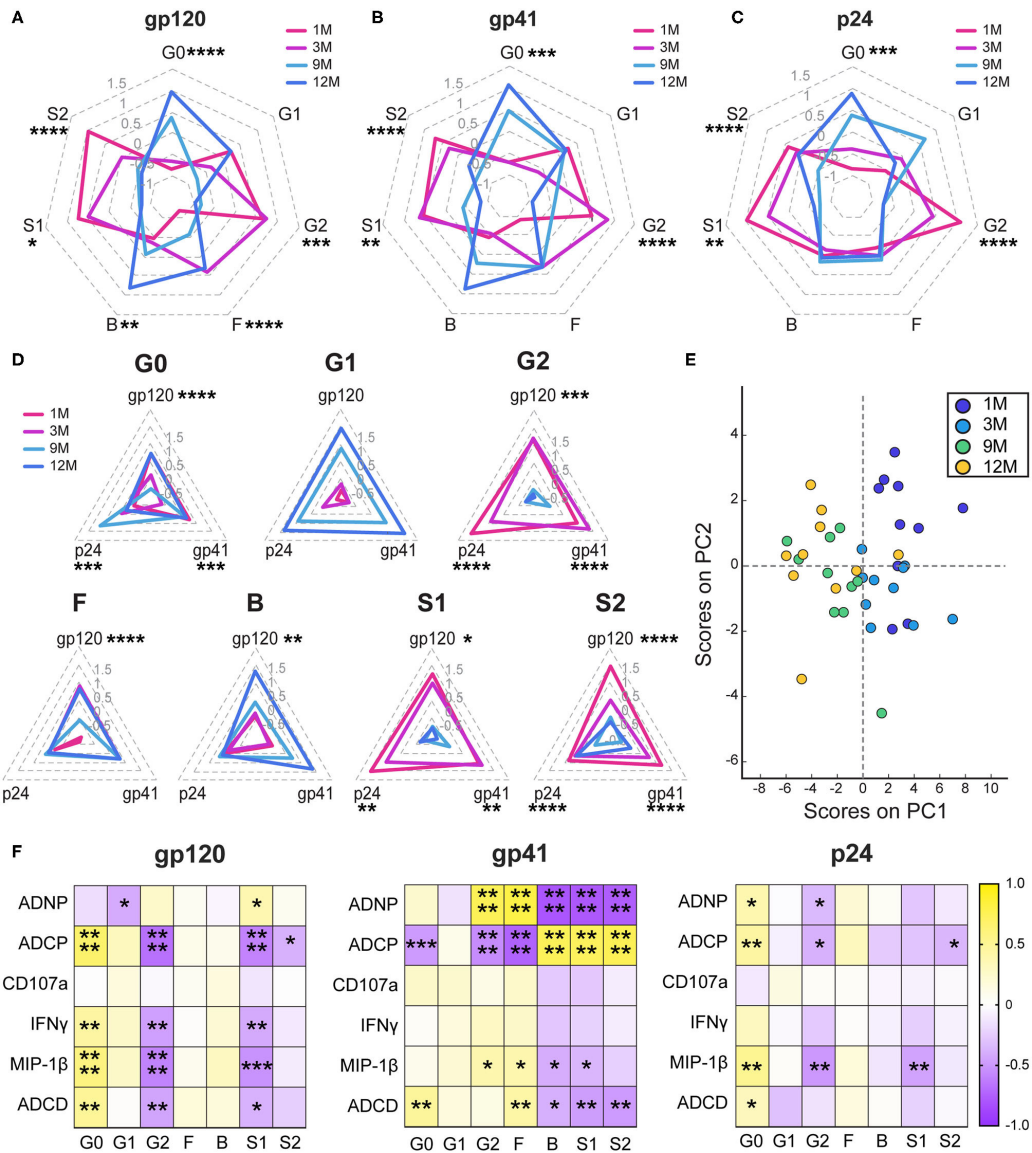
Antibody glycosylation changes following acute infection. **(A–C)** Radar plots depict the relative amounts of each monosaccharide on antigen-specific antibody Fc glycans. Glycans were determined by summing the percentages of each of 24 individual glycan peaks that display a given quality. G0, agalactosylated; G1, monogalactosylated; G2, digalactosylated; F, fucosylated; B, bisected with N-acetylglucosamine; S1, monosialylated; and S2, di-sialylated. Glycans percentages are normalized to an average of 0 and a standard deviation of 1 and the average of the 10 participants at each timepoint is graphed. Radars are labeled from 1.5 to −1 in gray. **(D)** Radar plots show the gp120-, gp41-, and p24- specific responses for each glycan attribute. Radars are labeled from 1.5 to −0.5 in light gray. **(E)** Principal components analysis of glycans was colored by timepoint. PC1 describes 50.4% of the variance and PC2 describes 11.4% of the variance. **(F)** Heatmaps show the r value of the Spearman correlation between function and glycan for all four timepoints. Statistics for radar plots evaluated as using one-way ANOVA. Heatmap generated with Spearman correlation. *p < 0.05, **p < 0.01, ***p < 0.01, and ****p < 0.001.

Given the dominant relationship of IgG1 and antibody effector function, we next aimed to define whether the IgG1s themselves evolved to gain function over the course of infection. Decades of research in the monoclonal therapeutics community have highlighted the key importance of Fc-glycosylation in shaping antibody IgG1 effector function (58–60). Thus, antigen-specific antibody Fc-glycosylation changes were analyzed using capillary electrophoresis (61). Changes in levels of agalactosylation (G0), monogalactosylation (G1), digalactosylation (G2), fucosylation (F), bisection with N-acetylglucosamine (B), monosialylation (S1), and disialylation (S2) were assessed for gp120- (Figure 2A), gp41- (Figure 2B), and p24-specific (Figure 2C) antibodies (Supplementary Figure 4). Consistent decreases in sialylated species and increases in agalactosylated inflammatory species were observed from early to late infection, and across multiple clinical states, as previously reported (26, 62, 63). However, those earlier studies had not assessed antibody N-glycosylation longitudinally with progression of disease. Antigen-specific variation was observed in the addition of bisected on gp120- and gp41-specific antibodies, but not on p24-specific antibodies. These changes were apparent when each glycan sugar was analyzed over time (Figure 2D), highlighting clear increases in agalactosylation and mono-galactosylation, whereas digalactosylation and sialylation decreased over time. Interestingly, more skewed changes occurred for fucosylation and bisection, increasing over time for gp120-specific but not p24- or gp41- specific antibodies (Figure 2D). Principal components analysis (PCA) highlighted the strikingly different evolutionary, antigen-specific, glycan patterns that emerged, which point to a dominant influence of disease progression on shaping antibody glycosylation (Figure 2E). Together these data highlight diverging evolutionary patterns for inflammatory and functional glycans that emerge with HIV disease progression.

To determine whether these glycan changes influenced the evolving differences in antibody effector function, we next examined the relationships between glycosylation and functional activity across antigen specificities (Figure 2F). As previously seen (64, 65), conserved relationships between inflammatory agalactosylation and less-inflammatory digalactosylation were observed with antibody function for gp120- and p24-specific responses (Figure 2F). Conversely, distinct relationships were observed for gp41-specific responses. Gp41-specific agalactosylation was linked to complement deposition, also modulated by fucosylation, bisection, and sialylation. Surprisingly, changes in neutrophil and monocyte phagocytosis were marked by inverse changes in galactosylation, sialylation, fucosylation, and bisection, highlighting potential antibody glycans that may be exploited naturally during infection to drive changes in antibody effector function at the antigen-specific level.

### Multivariate Signatures of Humoral Evolution

**Figure 3:**
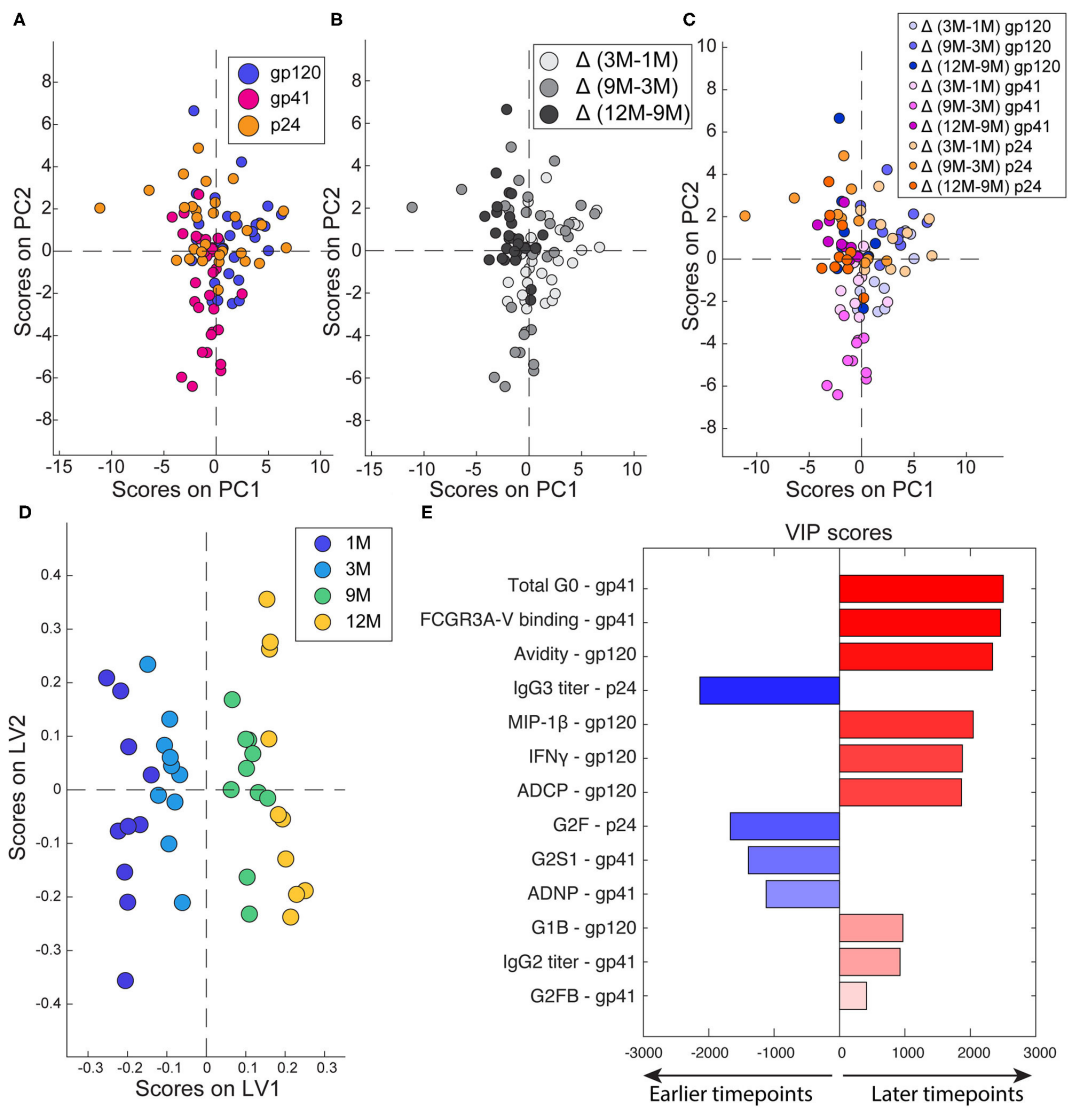
Multivariate determinants of antigen and timepoint. **(A–C)** To compare across timepoints and antigens, each antigen-specific response was treated as a separate point and deltas were calculated for 3M-1M, 9M-3M, and 12M-9M. Each dot represents one subject. **(A)** PCA was colored to show the separation of the different antigen-specific responses. PC1 captures 13.8% of the variance while PC2 captures 10.2% of the variance. **(B)** PCA colored to show the effect of timepoints. PC1 captures 13.8% of the variance. PC2 captures 10.2% of the variance. **(C)** PCA colored to show both timepoint (indicated by shade) and antigen (indicated by color). PC1 captures 13.8% of the variance and PC2 captures 10.2% of the variance. **(D)** A PLSR model was developed to define the features associated with timepoint in infection. Dots represent full responses, including measures from all three antigens, from one participant. Features associated with timepoint are captured on LV1, accounting for 44.2% of the variance. **(E)** Variable importance in the projection (VIP) scores are ordered, scaled, and colored according to their importance. Variables that point to the right are enriched in later timepoints, while those that point to the left are enriched in earlier timepoints.

Since robust changes in antibody effector functions, subclass, and glycosylation were observed over time, we next aimed to assess the differential effects of time and antigen-specificity on the composition and functionality of the evolving humoral immune response. An unbiased unsupervised PCA analysis was used to determine whether time or specificity had a greater effect in differentiating the antibodies. Each antigen-specific response was used as an individual data point, with antibody functionality, Fc receptor binding, subclass/isotype, avidity, and glycosylation, calculated as difference between timepoints, as input variables. The PCA highlighted the absence of any discernable differences due to antigen-specificities (Figure 3A). Conversely, when the PCA was colored by timepoint, significant separation between the timepoints was observed in antibody profiles (Figure 3B). Moreover, antigen-specificities tracked together over time (Figure 3C) highlighting the importance of timepoint as the primary driver of antibody differences.

Next, to define the specific changes that occurred in the functional humoral immune response over time, a supervised orthogonalized partial least squares regression (OPLSR) model was used across the four timepoints (Figures 3D,E). Variables including antigen-specific antibody subclass/class, FcR binding, glycosylation and functionality were included and the model was regressed on timepoints. As expected, robust separation was observed in antibody profiles over time, separated along latent variable 1 (LV1). The antibody features that contributed to this separation were then plotted on a variable importance scores plot (Figure 3E), where features in blue were present early in infection and those in red evolved over time. As expected, higher levels of p24-specific IgG3s were present in early infection, in addition to higher levels of digalactosylated gp41 antibodies able to recruit neutrophil phagocytosis. Conversely, functional maturation was observed over time in both gp41 and gp120-specific antibodies linked to increased inflammatory glycans. These data indicate that specific non-neutralizing functions, specifically those against gp120, mature across the first year of infection.

### Gp120-Specific Polyfunctionality Dictates Clinical Course

**Figure 4:**
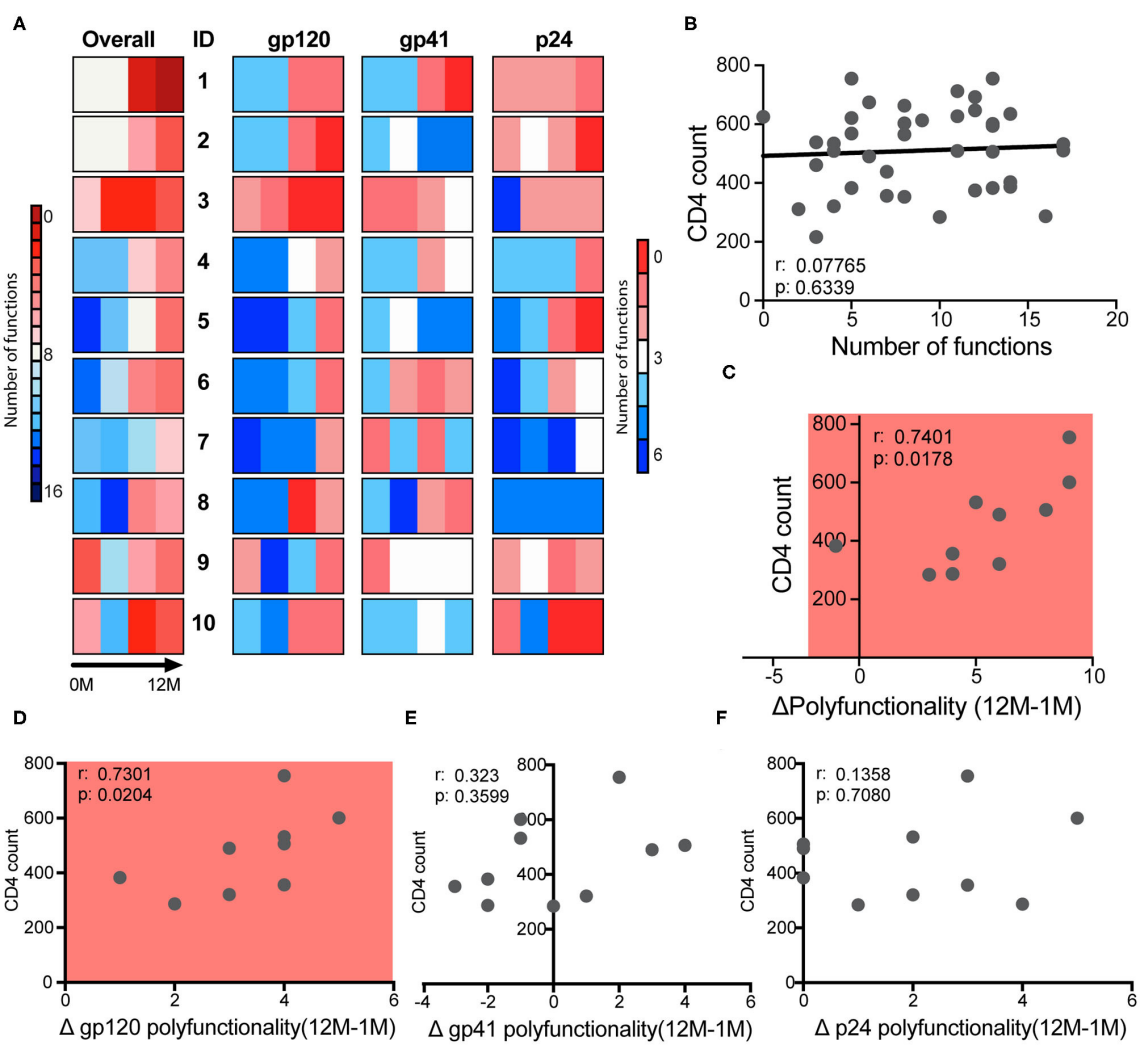
Polyfunctionality determines clinical course. **(A)** The bars show the polyfunctionality profiles for the 10 participants showing the number of functions each subject has overall, and for each antigen-specific response at each of four timepoints. **(B)** Correlation between the CD4 count and the number of functions that each participant had at each of four timepoints, with one dot per subject per timepoint. **(C)** Correlation between CD4 count at 12 months and the change in total polyfunctionality from month 1 to 12. **(D–F)** Correlation between CD4 count at 12 months and the change in antigen-specific functionality for gp120- **(D)**, gp41- **(E)**, and p24- **(F)** specific functions from month 1 to month 12. Correlations are Spearman correlations.

Early studies pointed to the predictive power of early ADCC levels and slower disease progression in a cohort of men (66). However, whether any specific antibody functions could predict slower disease progression in clade C infected women has yet to be defined. Thus, to begin to define whether any given antibody function, or group of functions were linked to disease progression, we examined overall differences in antibody effector profiles over time for the three antigen specificities (Figure 4A). Different patterns of antibody functionality were observed over time, in which some women exhibited many functions as early as the first month and high polyfunctionality (subjects 1–3). A group of women began with fewer antibody functions, but those functions increased modestly and steadily over infection (participants 4–7). Finally, a third group showed a decrease in polyfunctionality at 3 months of infection (subjects 8–10). These patterns were generally replicated for each of the antigen-specific responses, implying that subjects have a similar trajectory for their functional antibody evolution independent of antigen specificity. Importantly, one of the participants controlled their infection (sustained levels of viremia of <1,00 vc/mL in the absence of treatment) (subject 1) and exhibited high polyfunctionality at 1 month and robust polyfunctional antibody evolution over time. Subjects 2 and 3 also exhibited very similar profiles but were not controllers. Yet, subject 2 maintained high CD4 levels for almost 4 years before meeting clinical guidelines for care in South Africa (CD4 <350). In contrast, nearly all other subjects met the standards for care at around a year of follow-up. These data imply that polyfunctionality may be an important but not the only factor dictating viral control.

While no single function at any timepoint or over time was associated with markers of clinical progression, we next sought to determine whether combinations of functions or polyfunctionality, were associated with disease progression. Thus, the number of functions in response to all three antigens were summed for each subject over time, to generate a polyfunctionality score. No relationship was observed between CD4 counts and polyfunctionality at each timepoint (Figure 4B), even at the first time point, suggesting that early functions may not be a predictor of enhanced protection from disease progression in this cohort. Instead, the overall increase in polyfunctionality over the first year of infection (12M-1M) was significantly correlated with CD4 counts (Figure 4C), highlighting the presence of more functional antibodies in the setting of preserved CD4 cell function and help. Importantly, this relationship was largely driven by gp120-specific antibody polyfunctionality (Figures 4D–F), potentially pointing to a specific contribution by functional gp120-specific antibodies to viral control.

## Discussion

While the neutralizing antibody response takes several months to evolve, the functional, antibody Fc-mediated, humoral immune response to HIV matures rapidly following infection (1, 67). The responses to HIV antigens evolve with distinct kinetics, likely driven by the abundance of the antigen at different stages of infection as well as pre-existing cross-reactive antibodies within the B cell repertoire (1, 68). Specifically, gp41-specific humoral immunity appears earliest following infection, potentially drawing on pre-existing microbiome-cross reactive antibodies to emerge early (55, 56, 69). These responses are followed sequentially by p24-specific antibodies (8), and then by gp120-specific antibodies. Collectively, these mature over time, to ultimately confer autologous neutralization to historical viral variants after months of infection (1, 8). Furthermore, in a subset of infected individuals, these neutralizing responses evolve further to drive cross-neutralizing antibody activity that is thought to be key for desirable immune responses in future preventative vaccines (10, 70). However, despite some understanding of the evolution of the binding and neutralizing antibody response, little is known about the evolution of the functional humoral immune response across antigens following infection.

Here, with the advantage of longitudinal sampling over the course of the first year of infection, we observed a progressive evolution of the functional humoral immune response (Figure 1), shifting both in subclass/isotype selection profiles as well as in altered Fc-glycosylation (Figure 2). Interestingly, these changes evolved differentially depending on antigen specificity (Figure 3). Particularly, there were more extreme changes in the gp120-specific rather than the gp41- and p24-specific responses. In this cohort, nine of ten participants progressed, providing an opportunity to explore the longitudinal development of the antibody response and the relationship between early humoral immune responses and disease progression. Surprisingly, no single function across all three antigen-specificities tracked with improved clinical outcome (CD4 T cell counts). Instead, gp120-specific polyfunctionality was linked to reduced loss of CD4+ T cells (Figure 4), highlighting the critical importance of leveraging multiple innate immune cell functions to fully control the virus. These data highlight the importance of a coordinated gp120-specific response, rather than any single functional response, directed at the viral envelope, as a critical determinant of HIV viral control.

While our bead based assays provide highly reproducible and robust platforms to interrogate antibody functionality across individual antigens in a high-throughput manner, further experiments, probing actual viral uptake or infected-cell killing are warranted (42, 44, 46, 47). However, it is critical to note that these surrogate assays have been repeatedly linked to protection from SIV/SHIV infection (37, 38, 45, 71) and HIV disease progression (14, 16, 26), highlighting the importance of these antibody-measurements for HIV antiviral immunity. The analysis of individual antigen-specific functional evolution unfortunately necessitates the use of these bead based assays, as p24 is not available on the outside of viruses, and because it is difficult to control the level of gp41 expression on infected cells. Thus, while the assays used here do not necessarily represent the mechanistic basis by which these antibodies restrict HIV, the measurements clearly provide insights into the qualitatively evolution of the humoral immune response.

It is additionally critical to note that the assays performed here are poised to interrogate the evolution of IgG antibody effector function and glycosylation. Neutrophils constitutively expression the Fc-receptor for IgA (72), FcαR (CD89), providing a glimpse into IgA mediated function. Conversely, THP-1 cells and NK cells do not express the FcαR (73, 74). Furthermore, these cells differ in their constitutive Fc receptor expression, while monocytes constitutively express all Fc receptors (73), neutrophils constitutively express FcγRII, but must up-regulate FcγRI expression (73, 75). However, IgA responses also clearly evolved over time. Given the mucosal nature of early HIV infection, with replication largely taking place in the gastrointestinal system (76), the expanded use of mucosal innate immune cells may point to mechanisms by which antibodies may contribute to antiviral control in distinct immune compartments. Thus future studies, able to exploit mucosal innate immune cells that express FcαR, may have the capacity to fully interrogate the evolving immune response to HIV.

The exact contribution of antibody polyfunctionality to viral control is unclear. Whether polyfunctionality is a true driver of viral control or a biomarker of a qualitatively superior overall cellular/humoral immune response has yet to be determined. Polyfunctionality may however offer a multi-pronged attack on the virus. By targeting NK cells, complement, monocytes, and neutrophils to eliminate virus or virally infected cells, multiple back-up strategies exist to ensure the efficient removal of antibody opsonized viruses or cells, as viruses at different stages or sites of replication may be differently susceptible. Additionally, polyfunctionality may represent a biomarker for the evolution of broad Fc-receptor binding antibodies that may enable the delivery of antigens more efficiently to dendritic cells (DCs) to also drive more robust T cell immune responses, in a process termed the vaccinal effect (77). Thus, the ability to leverage multiple innate immune effector mechanisms simultaneously may provide the highest likelihood of control through multiple back-up lines of immune defense.

Strikingly different patterns of humoral evolution between gp120-, gp41-, and p24-specific responses were observed. Antibodies to gp41 showed a distinct immune trajectory, drawing on early cross-reactive IgM and IgA responses (55, 56, 78), to drive robust ADNP and ADCP as early as 1 month following infection. However, these antibodies show little functional development past 1 month. Previous studies have noted that gp41 specific immune responses, though they appear early, may be ineffective at restricting viral load during early infection (69). Distinct development trajectories were also noted for the p24-specific response, polarized toward NK activation early, then switching toward other functions, in the opposite direction to gp41-specific antibodies. Given our emerging appreciation for the potential importance of p24-specific responses in antiviral restriction (20) and enhanced dendritic cell opsonophagocytic control (22), these data point to distinct antibody effector functions that may be leveraged against p24. However, p24 is not expressed on the surface of the infected cell membrane or on the surface of the virion, pointing to other mechanisms by which this antibody specificity may contribute to antiviral restriction, including the induction of potential non-canonical functions such as TRIM21 (79).

The identification of immune correlates of viral control, particularly in acute infection, has critical implications for understanding HIV pathogenesis and ultimately the design of vaccines or therapeutics that may lead to the durable control of HIV. While no preventative vaccine or cure strategies have been successfully developed, at least two cases suggest that a “functional cure”–viral suppression in the absence of ART–may be achievable for periods of time (80–82). Monoclonal antibodies have emerged as tractable tools for the control and elimination of the HIV reservoir (83, 84). The data presented here provides new insights into the functional properties of antibodies that may confer enhanced control or elimination of infection. While further research is needed to determine whether and how polyfunctionality and specificity contribute to viral control and containment, there is substantial evidence for the importance of functional antibody responses in controlled HIV infection.

## Data Availability Statement

All datasets generated for this study are included in the article/Supplementary Material.

## Ethics Statement

The studies involving human participants were reviewed and approved by Partners IRB. The patients/participants provided their written informed consent to participate in this study.

## Author Contributions

MJ, JM, TN, and GA: conceptualization. MJ, JM, and GA: methodology. MJ and CB: formal analysis. MJ, JM, and CP: investigation. KD and TN: resources. MJ and GA: writing. KD, MA, TN, and GA: supervision. TN and GA: funding acquisition. All authors contributed to the article and approved the submitted version.

## Conflict of Interest

The authors declare that the research was conducted in the absence of any commercial or financial relationships that could be construed as a potential conflict of interest. The reviewer GL declared a past co-authorship with several of the authors MA and GA to the handling editor.
