# Supplementary material for: Tracking the Trajectory of Functional Humoral Immune Responses Following Acute HIV Infection
Source: Front Immunol. 2020 Aug 7;11:1744. doi: 10.3389/fimmu.2020.01744 (PMC7426367; doi:10.3389/fimmu.2020.01744)
Supplement: Supplementary file 2 [file Data_Sheet_2.docx]

Supplementary Material

# Supplementary Data File

**Full data.** The excel file contains the full data used for the study. **Col.** **A-C**. Participant identification. **Col D-E**. Clinical data. **Col F-AC.** Luminex data for the amount of antigen-specific antibodies for each class/subclass in media fluorescent intensity of PE. **Co. AD-AF. Antigen-specific antibody** avidity measured in avidity score. **Col. AG-AI.** Antigen-specific ADNP presented as phagocytosis score. **Col. AJ-AL.** Antigen-specific ADCP presented as phagocytosis score. **Co. AM-AU.** Antibody-dependent NK activation presented as percentage of cells NK cells positive for each of three markers (IFNγ, CD107a, MIP-1β). **Col AV-AX.** Antigen-specific ADCD presented as complement score. **Col AY-BV.** Gp120-specific antibody FC-glycosylation presented as percentage of total glycans. Glycans are named according to how many galactose residesu (G0-G2), presence of a fucose (F), presence of a bisecting GlcNac (B) and presence of sialic acid (S1-S2)**Col. BW-CD.** Summed totals for gp120-specific Fc glycans. Agalactosylation (G), monogalactosylation (G1), digalactosylation (G2), Fucosylation, presence of a bisecting N-Acetylglucosamine (Bisection), monosialylation (S1) and disialylation (S2). **Col. CE-DB.** P24-specific antibody Fc-glycosylation presented as percentage of total glycans. **Col DC-DJ.** Summed totals for gp120-specific Fc glycans. **Col. DK-EH.** Gp41-specific antibody Fc glycans presented as percentage of total glycans. **Col. EJ-EP.** Summed totals for p24-specific Fc glycans. **Col. EQ-HF.** Total IgG titers against a broad panel of antigens indicated in column title. **Col. HG- IN.** Total IgA titers of in response to panel of antigens. **Col. IO-JV.** C1q binding to antigen-specific antibodies. Antigen indicated in column title. **Col. JW-LD.** FcγIIa-H receptor binding to antigen-specific antibodies. Antigen indicated in column title. **Col. LE-ML.** FcγIIa-R receptor binding to antigen-specific antibodies. Antigen indicated in column title. **Col. MMM-NT.** FcγIIb receptor binding to antigen-specific antibodies. Antigen indicated in column title. **Col. NU-PB.** FcγIIIa-F receptor binding to antigen-specific antibodies. Antigen indicated in column title. **Col. PC-QJ.** FcγIIIa-V receptor binding to antigen-specific antibodies. Antigen indicated in column title. **Col. QK-RR.** FcγIIIb receptor binding to antigen-specific antibodies. Antigen indicated in column title.

# Supplementary Figures and Tables

## Supplemental Figure 1: Clinical course of infection.


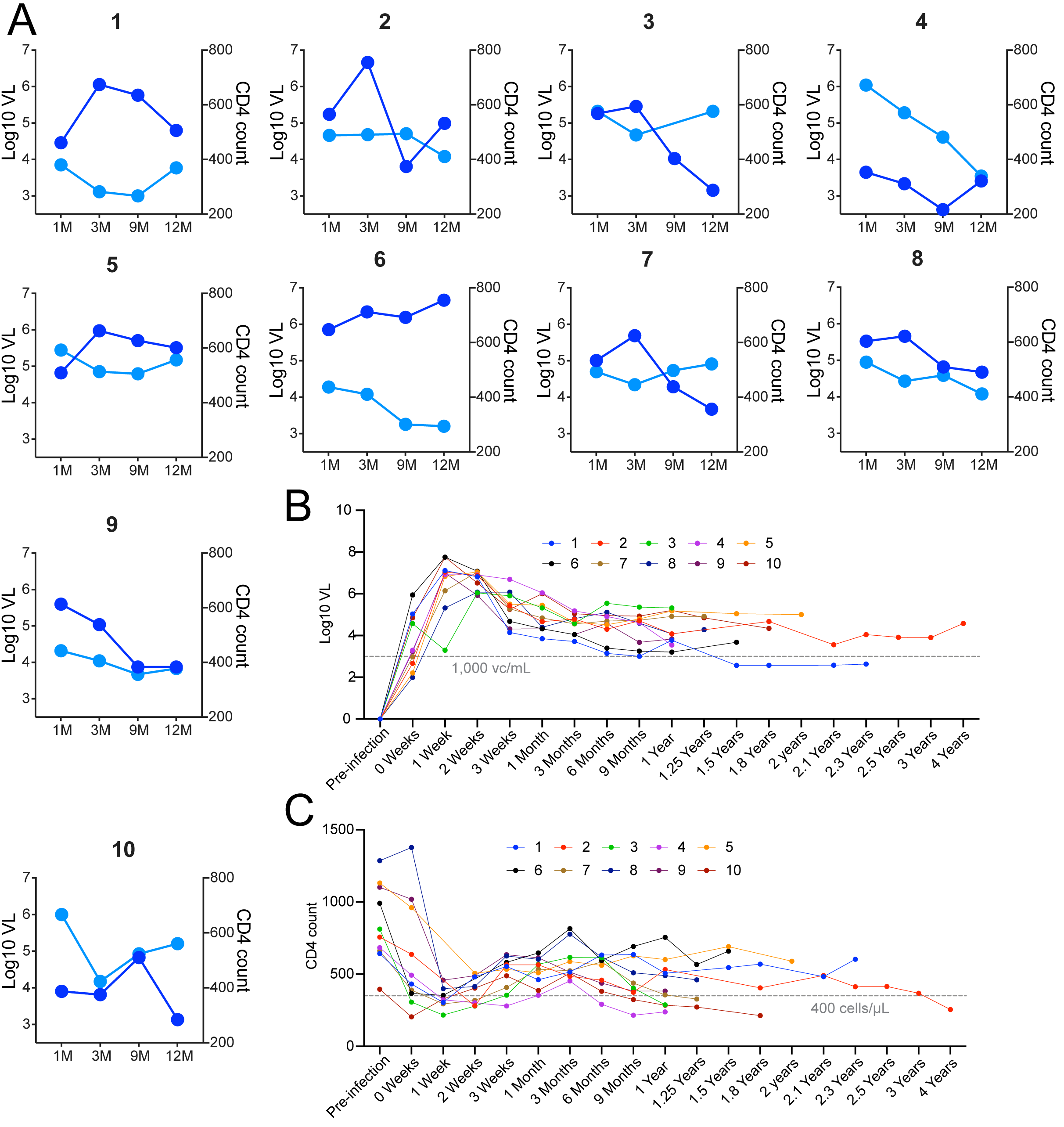


**Supplementary Figure 1.** **Clinical course of infection.** A. The viral load (light blue) and CD4 count (dark blue) for the ten participants is plotted for the first year of infection. B. Viral load is plotted for all participants for available timepoints from infection until initiation of anti-retroviral therapy. Dotted grey line is at 1,000 viral copies/mL, which defines viral control. C. CD4 count for all participants for available timepoints from infection until initiation of anti-retroviral therapy. Dotted grey line indicates 400 CD4 cells/μL, the national eligibility criteria for treatment at the time.

## Supplementary Figure 2: Non-neutralizing functions during acute infection


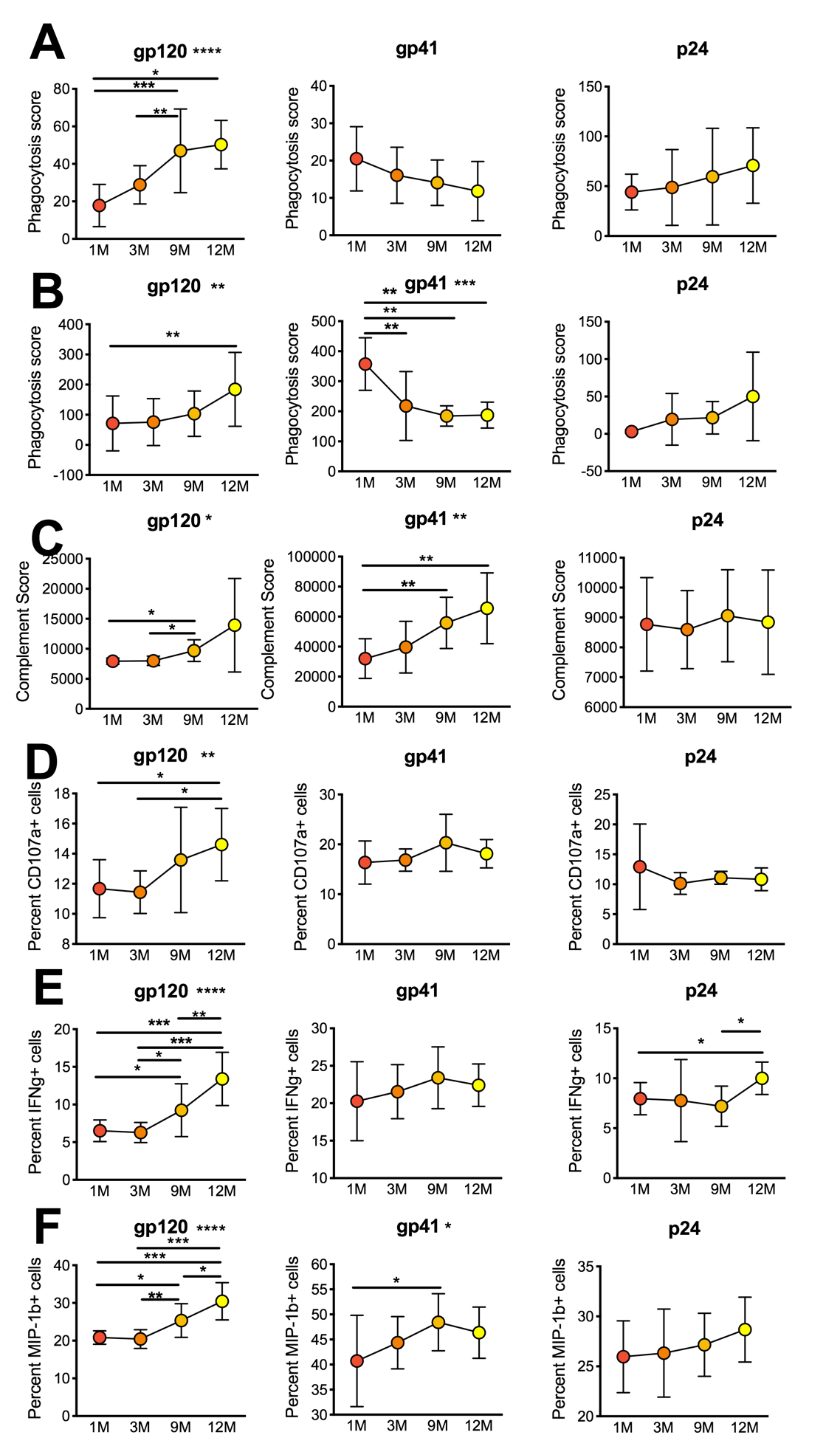


**Supplementary Figure 2. Non-neutralizing functions during acute infection.** The dot-line plots depict the average the average functionality for all 10 participants across the four timepoints for each antigen-specific function, ADCP (A)**,** ADNP (B)**,** ADCD (C) **and** NK**-mediated CD107a** (D)**, IFN**γ **(E),** and MIP-1β **(F)** expression. Dots are at average with standard deviation indicated Statistics for plots were evaluated using **RM** one-way ANOVA **with multiple comparisons**. *p<0.05, **p<0.01, ***p<0.01, ****p<0.001.

## Supplemental Figure 3: Antibody titer during acute infection

## Supplementary Figure 3. Antibody titer during acute infection. The dot-line plots depict the average the average antibody titer for all 10 participants across the four timepoints for each antigen-specific antibody class or subclass, IgG1 (A), IgG2 (B), IgG3 (C), IgG4 (D), Total IgG (E), IgA21(F), IgA2 (G), and IgM (H). Dots are at average with standard deviation indicated Statistics for plots were evaluated using RM one-way ANOVA. *p<0.05, **p<0.01, ***p<0.01, ****p<0.001.

## Supplemental Figure 4: Antigen-specific glycosylation changes during acute infection


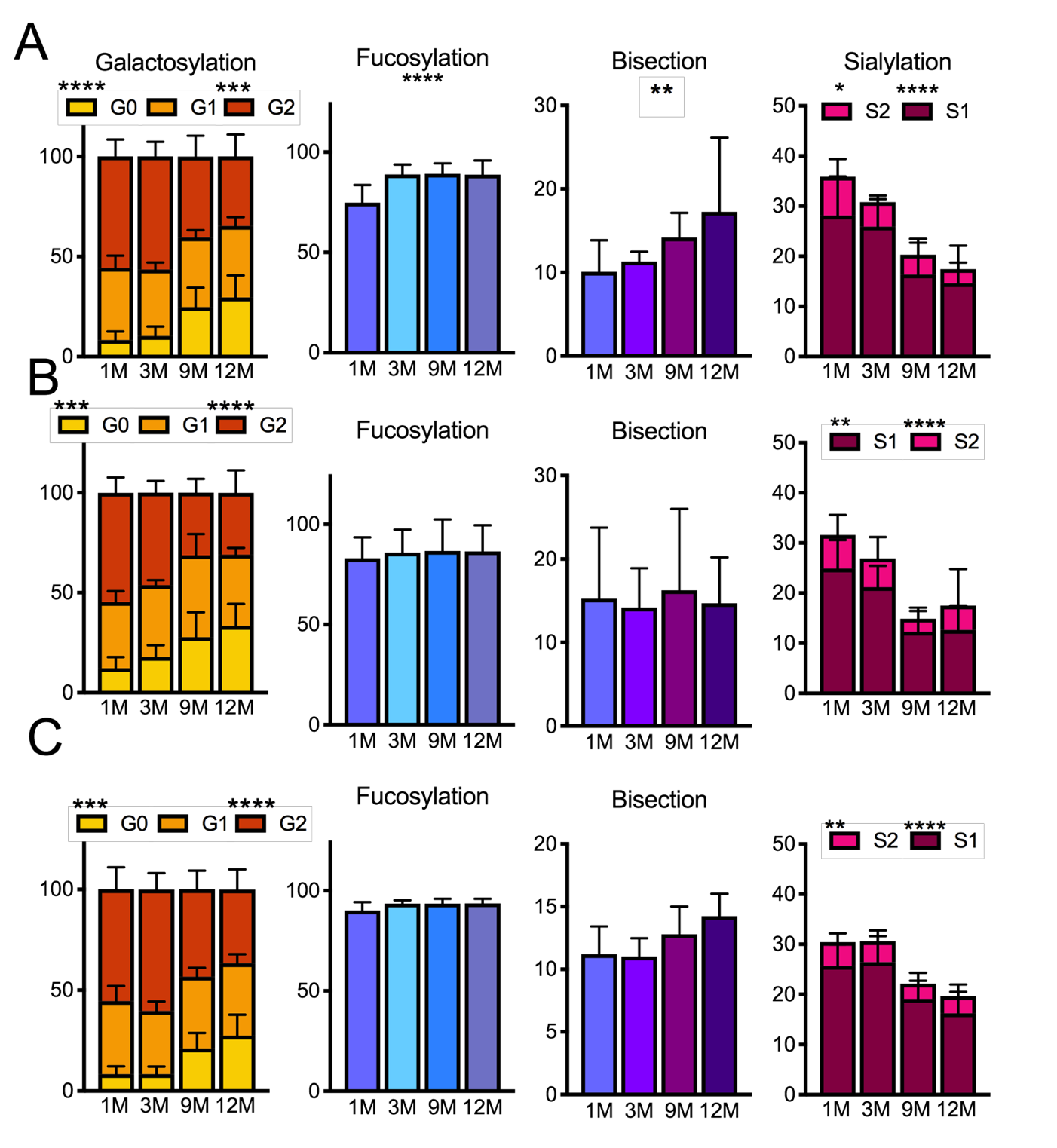


## Supplementary Figure 4. Antigen-specific glycosylation changes during acute infection. The bar plots depict the average of the different glycosylation attributes; Agalactosylation (G), monogalactosylation (G1), digalactosylation (G2), Fucosylation, presence of a bisecting N-Acetylglucosamine (Bisection), monosialylation (S1) and disalylation (S2) for all 10 participants. A. gp120-specific antibody glycosylation. B. gp41-specific antibody glycosylation. C. p24-specific antibody glycosylation. Bars at the average with standard deviation indicated. Statistics for plots were evaluated using one-way ANOVA. *p<0.05, **p<0.01, ***p<0.01, ****p<0.001.
